# Prevalence and clinical characteristics of lower limb atherosclerotic lesions in newly diagnosed patients with ketosis-onset diabetes: a cross-sectional study

**DOI:** 10.1186/1758-5996-6-71

**Published:** 2014-06-03

**Authors:** Mei-Fang Li, Ying Ren, Cui-Chun Zhao, Rong Zhang, Lian-Xi Li, Fang Liu, Jun-Xi Lu, Yin-Fang Tu, Wei-Jing Zhao, Yu-Qian Bao, Wei-Ping Jia

**Affiliations:** 1 Department of Endocrinology and Metabolism, Shanghai Jiao Tong University Affiliated Sixth People’s Hospital; Shanghai Diabetes Institute; Shanghai Clinical Center for Diabetes; Shanghai key Laboratory of Diabetes Mellitus, 600 Yishan Road, Shanghai 200233, China; 2 Department of VIP, Shanghai Jiao Tong University Affiliated Sixth People’s Hospital, 600 Yishan Road, Shanghai 200233, China

**Keywords:** Type 1 diabetes, Ketosis-onset diabetes, Type 2 diabetes, Lower limb arteries, Atherosclerosis

## Abstract

**Background:**

The clinical features of atherosclerotic lesions in ketosis-onset diabetes are largely absent. We aimed to compare the characteristics of lower limb atherosclerotic lesions among type 1, ketosis-onset and non-ketotic type 2 diabetes.

**Methods:**

A cross-sectional study was performed in newly diagnosed Chinese patients with diabetes, including 53 type 1 diabetics with positive islet-associated autoantibodies, 208 ketosis-onset diabetics without islet-associated autoantibodies, and 215 non-ketotic type 2 diabetics. Sixty-two subjects without diabetes were used as control. Femoral intima-media thickness (FIMT), lower limb atherosclerotic plaque and stenosis were evaluated and compared among the four groups based on ultrasonography. The risk factors associated with lower limb atherosclerotic plaque were evaluated via binary logistic regression in patients with diabetes.

**Results:**

After adjusting for age and sex, the prevalence of lower limb plaque in the patients with ketosis-onset diabetes (47.6%) was significantly higher than in the control subjects (25.8%, p = 0.013), and showed a higher trend compared with the patients with type 1 diabetes (39.6%, p = 0.072), but no difference was observed in comparison to the patients with non-ketotic type 2 diabetes (62.3%, p = 0.859). The mean FIMT in the ketosis-onset diabetics (0.73 ± 0.17 mm) was markedly greater than that in the control subjects (0.69 ± 0.13 mm, p = 0.045) after controlling for age and sex, but no significant differences were found between the ketosis-onset diabetics and the type 1 diabetics (0.71 ± 0.16 mm, p = 0.373), and the non-ketotic type 2 diabetics (0.80 ± 0.22 mm, p = 0.280), respectively. Age and FIMT were independent risk factors for the presence of lower limb plaque in both the ketosis-onset and non-ketotic type 2 diabetic patients, while sex and age in the type 1 diabetic patients.

**Conclusions:**

The prevalence and risk of lower limb atherosclerotic plaque in the ketosis-onset diabetes were remarkably higher than in the control subjects without diabetes. The features and risk factors of lower limb atherosclerotic lesions in the ketosis-onset diabetes resembled those in the non-ketotic type 2 diabetes, but different from those in the type 1 diabetes. Our findings provide further evidences to support the classification of ketosis-onset diabetes as a subtype of type 2 diabetes rather than idiopathic type 1 diabetes.

## Background

Ketosis-prone diabetes or atypical diabetes, manifested by remarkable insulin deficiency at diagnosis accompanied by unprovoked ketoacidosis without identifiable precipitating causes, and absence of islet-related autoantibodies, has been proposed as idiopathic or type 1B diabetes by the World Health Organization and the American Diabetes Association [1,2]. However, recent several studies, which suggested that patients with ketosis-prone diabetes possessed higher age of onset and BMI, ethnic-specific gene variants, and even a very variable response to diet and oral agents [3-6], have lent support to this idea that ketosis-prone diabetes is a subgroup of type 2 diabetes.

The comparisons of metabolic and immunologic characteristics among type 1, ketosis-prone and type 2 diabetes have been well established [7-9]. However, the prevalence and clinical characteristics of atherosclerosis and its complications are largely absent in ketosis-prone diabetes. In a previous study, we found that the prevalence and risk of carotid atherosclerosis in ketosis-onset diabetes were remarkably higher than those in subjects without diabetes, but resembled those in type 2 diabetes [10]. Furthermore, the features of carotid atherosclerotic lesions in ketosis-onset diabetes were also similar to those in type 2 diabetes [10]. However, because of a lack of type 1 diabetic group in our previous study, the differences of carotid atherosclerotic lesions could not be compared between type 1 and ketosis-onset diabetes.

In the current study, type 1 diabetes consisted of childhood-onset type 1 diabetes (≥17 years old) and latent autoimmune diabetes in adults (LADA), and the latter were frequently misdiagnosed as type 2 diabetes. In China, the former was infrequent (0.51 per 100,000) [11] while the latter was prevalent (5.9% for Chinese newly diagnosed type 2 diabetes) [12], and autoantibody (usually GAD antibody) is one of the most important factors to distinguish type 1 diabetes from type 2 diabetes [13]. The 53.4% of frequency of positive tests for anti-GAD was higher than the 25.8% frequency of positive IA-2 autoantibody in Chinese patients with type 1 diabetes [14]. In addition, the frequency of positive GAD or IA-2 antibodies was significantly higher in patients with type 1 diabetes than in those with type 2 diabetes [15-17].

It is generally accepted that accelerated atherosclerosis and its complications are typical features of type 2 diabetes [18,19]. For example, Scholte et al. [19] observed that the prevalence of coronary atherosclerosis reached to 80% even in asymptomatic type 2 diabetic patients. On the contrary, as the early-stage lesions of type 1 diabetes, diabetic microvascular complications, especially diabetic nephropathy, are main reasons for the morbidity and mortality in patients with type 1 diabetes reported by previous studies [20,21]. However, several recent studies found a higher prevalence of microvascular brain lesions in type 2 diabetics relative to non-diabetics and there was also a significant association between microvascular brain lesions and diabetes even after correction for other risk factors in type 2 diabetic patients [22,23]. Therefore, it is of great significance to discuss diagnostic classification of ketosis-prone diabetes from macroangiopathy angle.

Thus, one of the aims of the present study was to investigate the prevalence and features of lower limb atherosclerotic lesions in newly diagnosed Chinese patients with ketosis-onset diabetes. Secondly, we aimed to compare the characteristics of lower limb atherosclerotic lesions among control subjects, type 1 diabetes, ketosis-onset diabetes and non-ketotic type 2 diabetes. Finally, we intended to evaluate the risk factors for lower limb atherosclerotic plaque in the three diabetic groups.

## Methods

### Study population

This was a cross-sectional study and was partly based on the data from our previous studies [10,24]. The diagnostic criteria of newly diagnosed diabetes, diabetic ketosis, ketosis-onset diabetes and non-ketotic type 2 diabetes have been well-described in our previous study [10]. In addition, type 1 diabetes was defined as diabetes with positive GAD and/or IA-2 autoantibodies according to the standards of the World Health Organization [1].

Among 497 Chinese patients (≥17 years old) with newly diagnosed diabetes in our department between January 2007 and October 2008, 21 patients who did not meet above criteria and were absent of complete data were excluded (including two patients with positive islet-associated autoantibodies but without performing lower extremity ultrasonography). The other 476 patients were classified into three categories by above criteria. That was, 53 were diagnosed for type 1 diabetes, 208 for ketosis-onset diabetes and 215 for non-ketotic type 2 diabetes. Of these 53 patients with type 1 diabetes, 32 patients (60.4%) had isolated positive for GAD autoantibodies, 9 patients (17.0%) isolated positive for IA-2 autoantibodies, and 12 patients (22.6%) had combined positive for GAD and IA-2 autoantibodies. In addition, 62 control participants with a fasting plasma glucose (FPG) <6.0 mmol/L and a 2-h postprandial plasma glucose (2-h PPG) <7.8 mmol/L during a 75 g oral glucose tolerance test and with intact lower limb ultrasonography measurements were age-matched to patients with ketosis-onset diabetes.

The history of hypertension and cardio-cerebrovascular events (CCEs), alcohol consumption and smoking habits and medication use were obtained from all participants. The definitions of smoking and alcohol use, hypertension and CCEs were based on our previous studies [10,24,25]. The current uses of lipid-lowering drugs (LLDs), antihypertensive drugs (AHDs) and aspirin were recorded yes or no. The study was approved by the human research ethic committee of Shanghai Jiao Tong University Affiliated Sixth People’s Hospital, and written consent was obtained from all participants.

### Physical and laboratory examinations

The physical and laboratory examinations used in this study have been well-described in our previous studies [10,24]. In brief, we have conducted a systematic physical and biochemical test from all diabetic patients. Among these biochemical indicators, fasting and 2 hour C-peptide were used to assess pancreatic islet function of these patients, and the islet autoantibodies and ketosis situation were available to the diagnostic typing of diabetes. In addition, weight was divided into underweight (BMI < 18.5 kg/m^2^), overweight (23 to 24.9 kg/m^2^), and obesity (BMI > 25 kg/m^2^) based on Asia-Pacific criteria [26]. The glomerular filtration rate (GFR) was estimated based on serum creatinine concentration using the simplified MDRD formula: estimated GFR (eGFR) = 186.3 × (Serum creatinine)^‒ 1.154^ × (age)^‒ 0.203^ (×0.742 if female) [27]. The 24 h urinary albumin excretion (UAE) was determined as the mean of the values obtained from three separate early morning urine samples in the period of hospitalization. Urine ketones were collected from early morning urine sample and measured by Legal’s test [10]. The autoantibodies against GAD and IA-2 were measured by ELISA (Euroimmun Medizinische Labordiagnostika AG, Germany).

### Ultrasonography measurements

Color duplex ultrasonography was conducted by three trained, certified sonographers using a Acuson Sequoia 512 scanner (Siemens Medical Solutions, Mountain View, CA) with a 5–13 MHz linear transducer according to our previous methods [24,28]. Briefly, the study procedure involved scanning bilateral common femoral artery, profunda femoris artery, superficial femoral artery, popliteal artery, anterior tibial artery, posterior tibial artery, and peroneal artery for the presence of atherosclerotic plaque and stenosis. The femoral intima-media thickness (FIMT) on both sides was measured as the distance between the leading edge of the lumen-intima echo and the leading edge of the media-adventitia echo. Mean FIMT was defined as the mean values of bilateral FIMTs. Lower limb atherosclerotic plaque was defined as the presence of a focal structure encroaching into the arterial lumen of 0.5 mm or at least 50% greater than the thickness of the surrounding vessel wall or IMT of >1.5 mm in any of the above-mentioned lower limb arteries segments based on the Mannheim consensus [29]. Based on previous literatures [28,30-32], significant lower limb arterial stenosis was defined as luminal stenosis ≥ 50% and/or peak systolic velocity (PSV) ratio of ≥ 2.0 in at least one above-mentioned arteries. The reproducibility of measurements of lower limb atherosclerotic lesions has been reported in our previous study [28].

### Statistical analyses

The data were analyzed using SPSS 15.0 software. For continuous variables, normality was checked. If the data showed a normal distribution, variables were given as the mean ± S.D., and one-way ANOVA with LSD was used to determine differences among groups. If the data were not distributed normally, the Kruskal-Wallis test was employed and variables were expressed as median with interquartile range. For categorical variables, they were demonstrated by either absolute numbers or percentages. Chi-squared statistical analysis was used to evaluate the differences in categorical variables. Binary, multinomial logistic and linear regressions were applied to assess differences in categorical and continuous variables after controlling for age and/or sex. Spearman correlation was utilized to determine the intra-observer and inter-observer reproducibility. Binary logistic regression was performed to explore risk factors for lower limb atherosclerotic plaque. P < 0.05 (two-sided) was considered to be statistically significant. P < 0.10 (two-sided) was considered to be evident of statistical trends.

## Results

### Characteristics of the study subjects

The clinical characteristics of the studied subjects are manifested in Table 1. Both the type 1 and non-ketotic type 2 diabetes showed an equal sex distribution, whereas a strong male predominance was noted in the ketosis-onset diabetes, even after adjusting for age. Smoking, alcohol use, prevalence of hypertension and CCEs, rate of use of AHDs and aspirin, Body mass index (BMI), weight category, diastolic blood pressure (DBP), triglyceride (TC), high density lipoprotein cholesterol (HDL-C), FPG and 2 h PPG, fasting and 2 h C-peptide (FCP and 2 h PCP), and hemoglobin A1C (HbA1C) also displayed significant differences among the four groups after adjustment for age and sex (all p < 0.05).

**Table 1:** Characteristics of the study subjects

**Variables**	**Control subjects (n = 62)**	**Type 1 diabetes (n = 53)**	**Ketosis-onset diabetes (n = 208)**	**Non-ketotic type 2 diabetes (n = 215)**	***p *** **values**	* ***p *** **values**
Male (%)	43 (69.4%)	31 (58.5%)	153 (73.6%)	122 (56.7%)	0.002	0.030
Age (years)	47 ± 11	49 ± 18	49 ± 15	56 ± 14	<0.001	<0.001
Smoking (n, %)	38 (61.3%)	17 (32.1%)	84 (40.4%)	65 (30.2%)	<0.001	0.001
Alcohol (n, %)	40 (64.5%)	14 (26.4%)	37 (17.8%)	36 (16.7%)	<0.001	<0.001
Hypertension (%)	25 (40.3%)	11 (20.8%)	81 (38.9%)	98 (45.6%)	0.011	0.035
CCEs (%)	0 (0.0%)	2 (3.8%)	10 (4.8%)	31 (14.4%)	0.002	0.023
LLDs (%)	15 (24.2%)	10 (18.9%)	63 (30.3%)	70 (32.6%)	0.641	0.712
AHDs (%)	19 (30.6%)	8 (15.1%)	68 (32.7%)	95 (44.2%)	0.012	0.029
Aspirin (%)	8 (12.9%)	18 (34.0%)	66 (31.7%)	74 (34.4%)	0.009	0.013
BMI (kg/m^2^)	24.14 ± 3.67	22.34 ± 4.35	25.05 ± 3.54	25.16 ± 3.59	<0.001	<0.001
WHR	0.92 ± 0.07	0.89 ± 0.07	0.91 + 0.06	0.91 + 0.06	0.019	0.475
**Weight category					<0.001	<0.001
Underweight	3 (4.4%)	8 (15.1%)	3 (1.4%)	2 (0.9%)	——	——
Overweight	20 (32.3%)	6 (11.3%)	48 (23.1%)	49 (22.8%)	——	——
Obesity	22 (35.5%)	11 (20.8%)	97 (46.6%)	109 (50.7%)	——	——
SBP (mmHg)	124 ± 16	125 ± 17	127 ± 15	130 ± 17	0.042	0.146
DBP (mmHg)	78 ± 12	79 ± 10	81 ± 11	81 ± 9	0.101	0.023
*TG (mmol/l)	1.18 (0.88-1.92)	1.21 (0.84-1.55)	1.44 (1.00-2.19)	1.49 (1.08-2.21)	0.001	0.019
TC (mmol/l)	4.84 ± 1.02	4.83 ± 1.09	5.03 ± 1.22	4.82 ± 1.22	0.323	0.695
HDL-C (mmol/l)	1.28 ± 0.35	1.13 ± 0.26	1.05 ± 0.27	1.11 ± 0.25	<0.001	<0.001
LDL-C (mmol/l)	2.71 ± 0.88	3.24 ± 0.97	3.40 ± 1.03	3.12 ± 0.97	<0.001	0.075
*Cr (μmol/l)	71 (56–82)	59 (48–79)	68 (58–80)	67 (54–79)	0.036	0.442
*UAE (mg/24 h)	——	9.9 (7.1-18.1)	10.3 (6.5-23.8)	8.9 (6.4-24.8)	0.823	0.917
*eGFR (ml/min/1.73 m^2^)	103 (93–116)	109 (89–136)	112 (92–134)	98 (82–118)	0.432	0.588
*FPG (mmol/l)	4.96 (4.67-5.24)	7.99 (6.02-11.44)	9.74 (7.63-12.45)	7.53 (6.39-9.67)	<0.001	<0.001
*2 h PPG (mmol/l)	5.84 (5.04-6.58)	14.66 (10.49-19.61)	16.66 (12.94-20.79)	13.14 (10.39-16.66)	<0.001	<0.001
*FCP (ng/mL)	2.05 (1.6-3.03)	0.6 (0.3-0.84)	1.23 (0.74-1.92)	2.02 (1.36-2.93)	<0.001	0.030
*2 h C-P (ng/mL)	7.99 (6.56-9.92)	0.92 (0.62-1.77)	2.31 (1.55-3.66)	4.65 (3.12-6.35)	<0.001	0.004
HA1C (%)	5.3 ± 0.34	12.39 ± 2.36	11.83 ± 2.05	9.81 ± 2.58	<0.001	<0.001

Values are presented as mean ± SD, median with interquartile range, or percentages. *Non-normal distribution of continuous variables. **Weight was divided into underweight (BMI < 18.5 kg/m^2^), overweight (23 to 24.9 kg/m^2^), and obesity (BMI > 25 kg/m^2^) based on Asia-Pacific criteria.

P value: The p-values were not adjusted for age and sex for the trend.

*P value: The *p value were adjusted by age and sex for the trend.

### Comparison of lower limb atherosclerotic lesions

**Figure 1:**
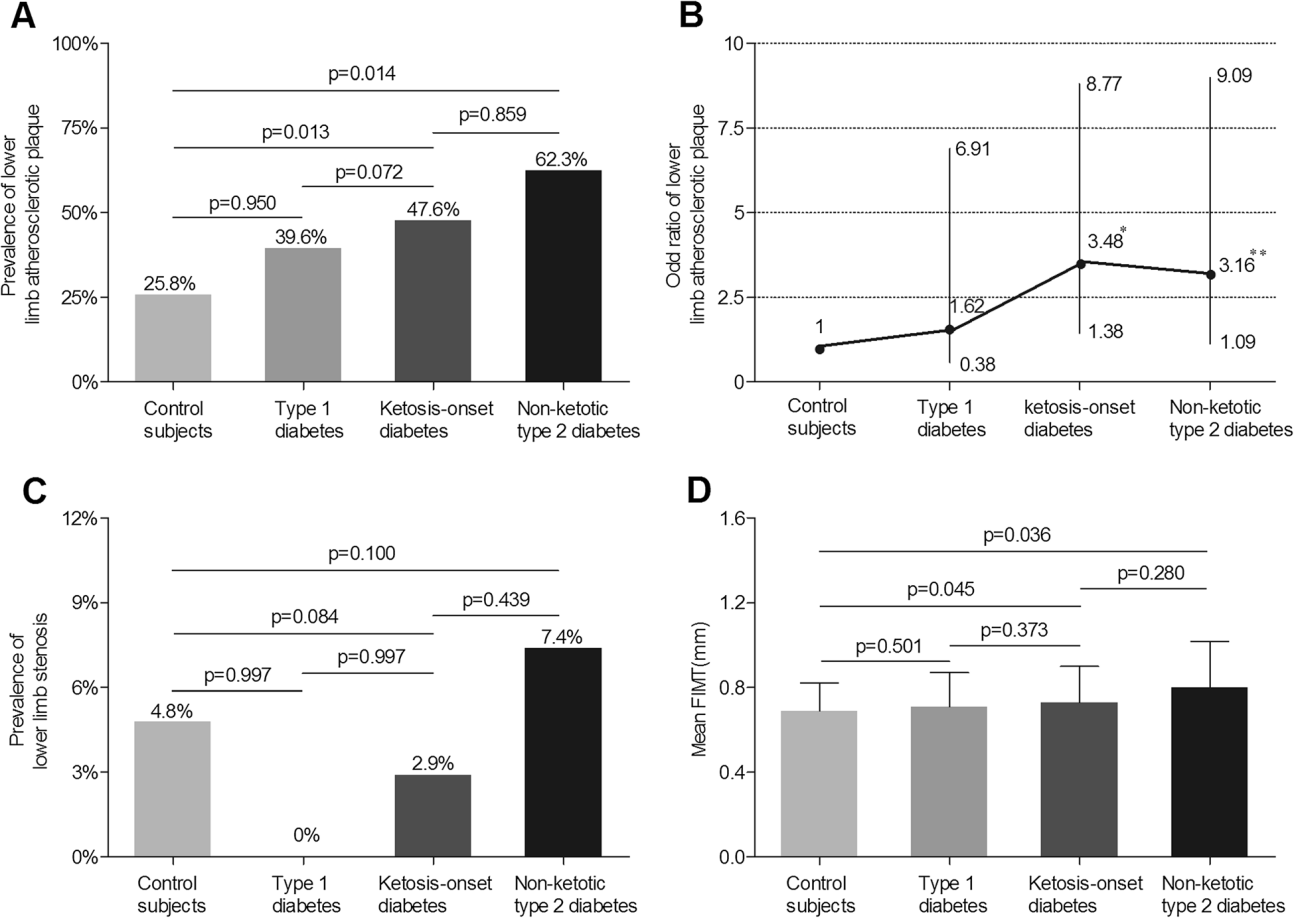
**Comparison of lower limb atherosclerotic lesions among the four groups after adjusting for age and sex. (A)** Comparison of the prevalence of lower limb atherosclerotic plaque among the four groups. The p-value for four group comparisons was 0.013. **(B)** Odd ratio of lower limb atherosclerotic plaque for the type 1, ketosis-onset and non-ketotic type 2 diabetic subjects in comparison to control subjects without diabetes. The bars represent the 95% confidence interval. Compared with control subjects, where *p = 0.002 and **p = 0.041. **(C)** Comparison of the prevalence of lower limb stenosis among the four groups. The p-value for four group comparisons was 0.167. **(D)** Comparison of the mean FIMT among the four groups. The p-value for four group comparisons was 0.015.

Figure 1 demonstrates the comparisons of lower limb atherosclerotic lesions among the four groups after adjustment for age and sex. The prevalence of lower limb atherosclerotic plaque in the ketosis-onset diabetic group was significantly higher than that in the control group (p = 0.013), and had a higher trend compared with the type 1 diabetic group (p = 0.072), but no significant difference was observed in comparison to the non-ketotic type 2 diabetic group (p = 0.859), respectively (Figure 1A). The odds ratio of lower limb atherosclerotic plaque in the ketosis-onset diabetes was the highest among the four groups, using the control group as a reference [OR, 3.48 (95% CI, 1.38-8.77) for the ketosis-onset diabetes; OR, 1.62 (95% CI, 0.38-6.91) for the type 1 diabetes; OR, 3.16 (95% CI, 1.10-9.09) for the non-ketotic type 2 diabetes]. The odds ratio of lower limb atherosclerotic plaque exhibited difference between the ketosis-onset and type 1 diabetes (p = 0.062), but no difference between the ketosis-onset and non-ketotic type 2 diabetic patients (p = 0.613) (Figure 1B).Contrary to lower limb atherosclerotic plaque, there was no statistical significance in the prevalence of lower limb stenosis among the control group (4.8%), the type 1 diabetic group (0%), the ketosis-onset diabetic group (2.9%) and non-ketotic type 2 diabetic group (7.4%) after controlling for age and sex (p = 0.167) (Figure 1C).Similar to lower limb atherosclerotic plaque, the mean FIMT in the ketosis-onset diabetes (0.73 ± 0.17 mm) was markedly higher than that in the control subjects (0.69 ± 0.13 mm, p = 0.045), but no significant differences were found among the ketosis-onset diabetes, type 1 diabetes (0.71 ± 0.16 mm, p = 0.373), and non-ketotic type 2 diabetes (0.80 ± 0.22 mm, p = 0.280) after controlling for age and sex (Figure 1D).

### Comparison of lower limb atherosclerotic lesions stratified by sex and age in each diabetic group

**Figure 2:**
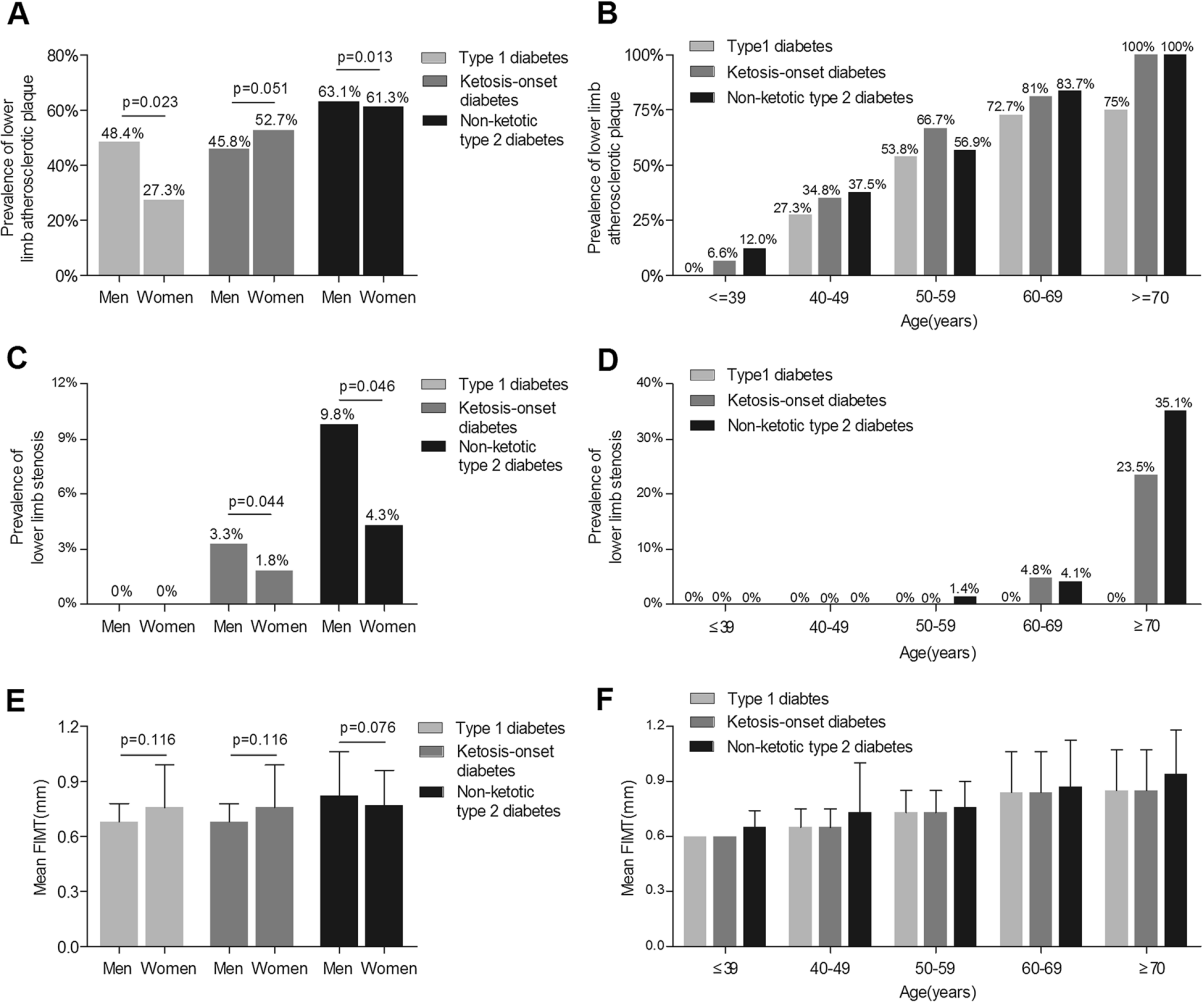
**Comparison of lower limb atherosclerotic lesions stratified by sex and age in diabetics. (A)** The prevalence of lower limb atherosclerotic plaque stratified by sex in the type 1, ketosis-onset and non-ketotic type 2 diabetic subjects after adjusting for age. **(B)** The prevalence of lower limb atherosclerotic plaque stratified by age in the type 1, ketosis-onset and non-ketotic type 2 diabetic subjects after adjusting for sex. The p-values for group comparisons were successively 0.001, <0.001 and <0.001 in the type 1 diabetic subjects, ketosis-onset and non-ketotic type 2 diabetic subjects. **(C)** The prevalence of lower limb stenosis stratified by sex in the type 1, ketosis-onset and non-ketotic type 2 diabetic subjects after adjusting for age. **(D)** The prevalence of lower limb stenosis stratified by age in the type 1, ketosis-onset and non-ketotic type 2 diabetic subjects for sex. The p-values for group comparisons were 0.001 and <0.001 in the ketosis-onset and non-ketotic type 2 diabetic subjects, respectively. **(E)** Comparison of mean FIMT stratified by sex in the type 1, ketosis-onset and non-ketotic type 2 diabetic subjects after adjusting for age. **(F)** Comparison of mean FIMT stratified by age in the type 1, ketosis-onset and non-ketotic type 2 diabetic subjects after adjusting for sex. The p-values for group comparisons were all <0.001 in type 1 diabetic subjects and ketosis-onset and non-ketotic type 2 diabetic subjects.

Analyses of lower limb atherosclerotic lesions stratified by sex and age in each diabetic group are showed in Figure 2. A significant sex-related difference and a remarkable increase with age were found in the prevalence of lower limb atherosclerotic plaque in each diabetic group (Figure 2A, B). Interestingly, the prevalence of lower limb atherosclerotic plaque in the ketosis-onset diabetes was higher in female than in male after adjustment for age, opposite to the type 1 and non-ketotic type 2 diabetes. The prevalence of lower limb stenosis also demonstrated significant sex- and age-related differences in the ketosis-onset and non-ketotic type 2 diabetic groups, rather than in the type 1 diabetic group (Figure 2C, D). The mean FIMT remarkably increased with age, but had no statistical significance between sexes in each subgroup (Figure 2E and F).

### Comparison of the mean FIMT between the subjects with and without lower limb plaque in each diabetic group

**Figure 3:**
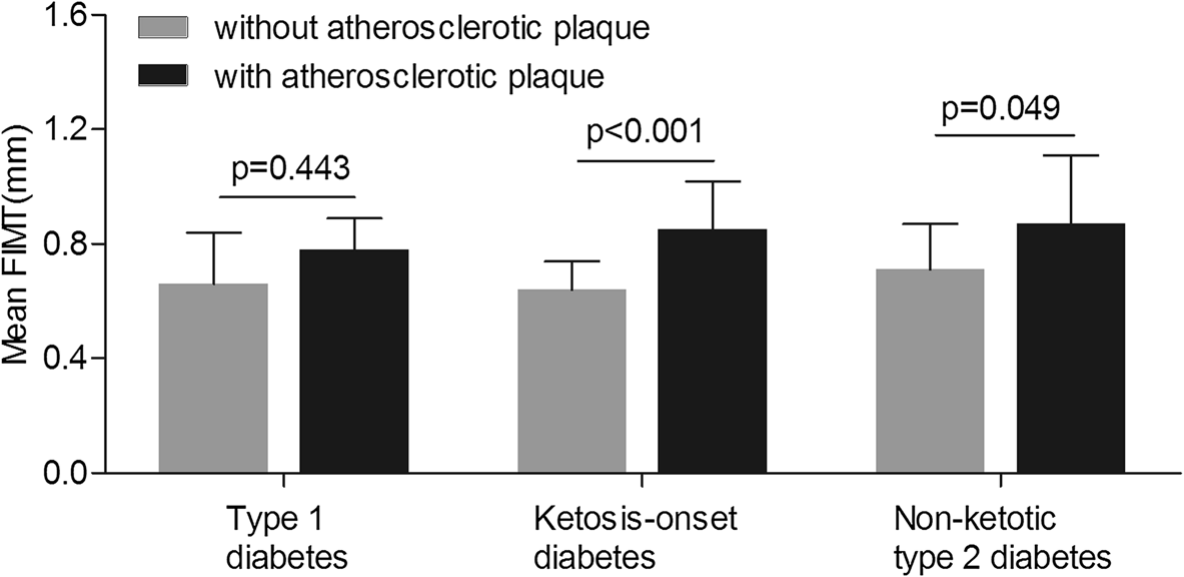
Comparison of the mean FIMT in subjects with and without lower limb atherosclerotic plaque in each diabetic group.

Figure 3 compares the mean FIMT value of the subjects with and without lower limb atherosclerotic plaque after controlling for age and sex in each diabetic group. In both the ketosis-onset (0.64 ± 0.10 mm and 0.85 ± 0.17 mm for the subjects without and with lower limb atherosclerotic plaque, respectively, p < 0.001) and non-ketotic (0.71 ± 0.16 mm and 0.87 ± 0.24 mm for the subjects without and with lower limb atherosclerotic plaque, respectively, p = 0.049) diabetic groups, the mean FIMT value was markedly higher in the subjects with lower limb atherosclerotic plaque than those without lower limb atherosclerotic plaque. In contrast, there was no significant difference in the mean FIMT value between the type 1 diabetic patients without and with lower limb atherosclerotic plaque after controlling for age and sex (0.66 ± 0.18 mm and 0.78 ± 0.11 mm, respectively, p = 0.443).

### Analyses of risk factors for lower limb atherosclerotic plaque in diabetic patients

The risk factors for lower limb atherosclerotic plaque were performed by binary logistic regression analyses after adjusting for the variables in Table 1. In the type 1 diabetes, sex (women) [Exp (B), 17.03 (95% CI, 1.28-26.88), p = 0.03] and age [Exp (B), 1.35 (95% CI, 1.07-1.69), p = 0.01] were significantly associated with lower limb atherosclerotic plaque. In the non-ketotic type 2 diabetes, age [Exp (B), 1.12 (95% CI, 1.07-1.18), p < 0.001] and mean FIMT [Exp (B), 1.01 (95% CI, 1.00-1.01), p = 0.013] were independent risk factors for lower limb atherosclerotic plaque. Interestingly, the ketosis-onset diabetic patients had the same risk factors for lower limb atherosclerotic plaque [Exp (B), 1.20 (95% CI, 1.11-1.30) for age, p < 0.001; Exp (B), 1.04 (95% CI, 1.02-1.06) for mean FIMT, p < 0.001] as the non-ketotic type 2 diabetes. For all diabetic patients, sex (women) [Exp (B), 2.13 (95% CI, 1.12-4.08), p = 0.022], age [Exp (B), 1.15 (95% CI, 1.11-1.20), p < 0.001] and mean FIMT [Exp (B), 1.01 (95% CI, 1.005-1.014), p < 0.001] were risk factors for lower limb atherosclerotic plaque.

## Discussion

The present study showed that the atherosclerotic characteristics of the ketosis-onset diabetes were similar to those of the non-ketotic type 2 diabetes rather than the type 1 diabetes, which provided powerful clinical evidences for the classification of ketosis-onset diabetes from the point of macrovascular lesions.

The immunology and pathophysiology of ketosis-onset diabetes have been sufficiently studied by other investigators [7,8]. However, data were limited regarding macrovascular complications in ketosis-onset diabetes. Although we previously have compared features of carotid atherosclerosis between ketosis-onset diabetes and non-ketotic type 2 diabetes [10], it is not entirely clear whether similar atherosclerotic features exhibited between ketosis-onset and type 1 diabetes, due to a lack of type 1 diabetic group in our previous study. Therefore, we carried out the present study to comprehensively compare atherosclerotic characteristics among type 1, ketosis-onset and non-ketotic type 2 diabetes and to further investigate whether similar atherosclerotic changes between ketosis-onset diabetes and non-ketotic type 2 diabetes are a common phenomenon in other arteries, not just in carotid arteries. To the best of our knowledge, this is the first time to systematically compare atherosclerotic lesions among type 1, ketosis-onset, and non-ketotic type 2 diabetes.

Consistent with previous reports [7,8,33], our results showed the proportion of overweight or obesity as well as BMI value were almost identical in the ketosis-onset and non-ketotic type 2 diabetes, but were remarkably higher compared with the type 1 diabetes. The levels of FCP and 2 h PCP in the patients with ketosis-onset diabetes was intermediate between those with type 1 and type 2 diabetes, partly explained by the fact that ketosis-onset diabetes has a predominant insulin secretory defect rather than a decline in beta-cell mass months after recovery from the index episode of ketoacidosis [4,34,35]. FPG and 2 h PPG in the ketosis-onset diabetes were higher than in the type 1 and non-ketotic type 2 diabetes, which suggested that the impairment of beta-cell was acute in ketosis-onset diabetes [36]. The exact mechanisms of transient beta-cell failure in ketosis-onset diabetes have not been clarified yet, but several lines of evidence indicated a genetic propensity to glucotoxicity- or glucolipotoxicity-induced oxidative stress might be responsible for transient beta-cell function [37,38]. For example, Sobngwi et al. [39] found ketosis-prone type 2 diabetes was associated with low glucose-6-phosphate dehydrogenase activity, an intracellular enzyme essential to defense mechanisms against oxidative stress.

In addition, our study found that the prevalence of ketosis-onset diabetes is approximately 50% in Chinese patients with newly diagnosed diabetes, though the incidence of that is unknown for now. The possible reason that nearly half of newly diagnosed diabetic subjects had ketosis-onset is that the illness of patients with ketosis-onset diabetes tended to be more serious; thus patients with ketosis-onset diabetes are more likely to be hospitalized.

In keep with features of carotid plaque of ketosis-onset diabetes in our previous study [10], the prevalence of lower limb atherosclerotic plaque in the ketosis-onset diabetic patients was also remarkably higher relative to the subjects without diabetes and also significantly increased with age. In accordance with our results, Danese et al. [40] demonstrated that diabetes was significantly associated with the presence of atherosclerotic plaques in lower limb district in a retrospective study. Rajala et al. [41] revealed that the prevalence of atheromatous plaques in femoral arteries was 77% in elderly Finns with diabetes mellitus or impaired glucose tolerance, close to our previous study [24], which clarified that elderly patients had a higher prevalence of lower limb atherosclerosis than younger patients in Chinese type 2 diabetes. Moreover, taking the subjects without diabetes as a reference, the 3.48-fold risk of lower limb atherosclerotic plaque in the ketosis-onset diabetes came near the non-ketotic type 2 diabetes, but higher than the type 1 diabetes, possibly due to later-onset age, higher BMI, and higher blood pressure, lipid and glucose levels for the ketosis-onset diabetes versus type 1 diabetes. For the ketosis-onset diabetes, the prevalence and risk of lower limb atherosclerotic plaque were higher than that of carotid atherosclerotic plaque we previously reported [10], explained by the fact that atherosclerotic lesions were more frequent in femoral arteries than carotid arteries independent of increasing number of risk factors [42,43]. Unlike no sex difference in the prevalence of carotid atherosclerotic plaque [10], female with ketosis-onset diabetes had a much higher prevalence of lower limb atherosclerotic plaque than male. Amusingly, contrary to gender feature of lower limb atherosclerotic plaque in the ketosis-onset diabetes, the type 1 and non-ketotic type 2 diabetes in the present study had higher lower limb atherosclerotic plaque in male than in female as shown by previous studies [24,44]. The reason for this sex-discrepancy between the ketosis-onset diabetes and type 1 and type 2 diabetes may attribute to strong male predomination in the ketosis-onset diabetes compared with an equal ratio of sex in the type 1 and type 2 diabetes, which suggested that the ketosis-onset diabetes possessed its own characteristics in lower limb atherosclerotic lesions, even though most of lower limb atherosclerotic features in the ketosis-onset diabetes were approximately same as the non-ketotic type 2 diabetes. Considered detection of lower limb atherosclerotic plaque by ultrasound as a subclinical indicator of cardiovascular events [45-47], early detection and adequate treatment of atherosclerosis are beneficial to the prevention of future cardiovascular events in ketosis-onset diabetes.

Similar to the prevalence of carotid stenosis of ketosis-onset diabetes in our previous study [10], no obvious difference was observed in the prevalence of lower limb stenosis among the four groups. Consistent with our findings, two previous studies [48,49] reported that no significant differences were found between the patients with and without diabetes in the percentage of stenosis of below-knee arteries. The reason for this may be that stenosis was the late-stage vascular dysfunction of diabetes, whereas our study subjects were newly diagnosed diabetes, so the prevalence of stenosis in our study was relatively low, contributing to no different prevalence of stenosis among different groups. Different from the prevalence of carotid stenosis without sex and age differences in ketosis-onset diabetes [10], the prevalence of lower limb stenosis was remarkably higher in men than in women and increased with age in both the ketosis-onset and non-ketotic type 2 diabetes, but not in the type 1 diabetes. American Diabetes Association stated that the risk of peripheral arterial disease increases by age in diabetic patients [50]. For example, Tseng [51] demonstrated that older age was an independent risk factor for peripheral arterial obstructive disease in Taiwanese patients with type 2 diabetes. The sex- and age-related differences of lower limb stenosis between the type 1 diabetes and ketosis-onset and type 2 diabetes may be that the numbers of subjects were relatively small (53 patients) and there were no patients with lower limb stenosis in the type 1 diabetic group. Given that lower limb stenosis is associated with the extent and prognosis of coronary artery disease [52], the appearance of lower limb stenosis can be served as a prognostic indictor of coronary artery disease in ketosis-onset diabetes.

The mean CIMT of ketosis-onset diabetes in our previous study [10] was significantly higher than control subjects and remarkably related to age, but not sex. Additionally, the patients with carotid plaque had remarkably a higher mean CIMT than those without carotid plaque for ketosis-onset diabetes. The mean FIMT of the ketosis-onset diabetes in our current study had the same features as the mean CIMT in our previous study. Consistent with our results, Kogawa et al. [53] reported that the mean FIMT of subjects with type 2 diabetes mellitus was remarkably higher than those without diabetes. Kirhmajer et al. [54] reported that FIMT in patients with coronary artery disease was increased compared with those without coronary artery disease, and triglycerides, BMI, male gender and smoking was an independent risk factor for increased FIMT. No sex-related differences in the FIMT of ketosis-onset diabetes can be explained by the Bogalusa heart study [55], which found that age-adjusted FIMT showed gender differences only among young adult whites rather than blacks and suggested that ethnic differences might be major contributors to no sex-related differences in the FIMT. Seeing that increased FIMT can predict extent and severity of coronary artery disease [56,57], the usefulness of FIMT can be used for assessing the outcomes of coronary artery disease in ketosis-onset diabetes.

The mean FIMT existed significant difference between the patient without and with lower limb plaque in the ketosis-onset and non-ketotic type 2 diabetic subjects, rather than in the type 1 diabetic subjects. We cannot successfully explain this finding. A possible explanation for this observation may be that ketosis-onset diabetes and type 2 diabetes shared the similar pathogenesis of atherosclerosis.

In keep with risk factors of carotid plaque of the ketosis-onset diabetes in our previous study [10], age and mean FIMT were independent risk factors for the presence of lower limb plaque in the ketosis-onset diabetes. More interestingly, risk factors of lower limb plaque in the ketosis-onset diabetes were the same as those in the non-ketotic type 2 diabetes, but not the type 1 diabetes, which indirectly supported the idea for classifying ketosis-onset diabetes as a subtype of type 2 diabetes.

Our study has important clinical implications. First, the general clinical features and macrovascular complications in ketosis-onset diabetes were more similar to type 2 diabetes rather than type 1 diabetes, whereas there were also some similar features between ketosis-onset diabetes and type 1 diabetes. Therefore, our study provided powerful evidences for the hypothesis that diabetes is a heterogeneous disease [58]. Secondly, there was a higher prevalence of atherosclerotic plaque in ketosis-onset diabetes than normal controls, so early screening and intervention were beneficial to reduce future cardiovascular events. Finally, due to ketosis-onset diabetes more inclined to type 2 diabetes, the clinical treatments of ketosis-onset diabetes should be similar to those of type 2, but not type 1 diabetes. For example, the individuals with ketosis-onset diabetes are able to maintain optimal glycemic control on oral antidiabetic agents after recovering from the initial crisis with insulin therapy [8].

There are several limitations to our study. First, the cross-sectional design restricted our ability to assess the evolutionary process of atherosclerotic lesions. Further prospective studies are needed to clarify the characteristics of atherosclerotic development in ketosis-prone diabetes. Secondly, the age of the type 1 diabetic subjects in our study was older than usual. However, because atherosclerosis is closely related to age, our study excluded the patients with diabetes aged less than 17 years old. The patients with type 1 diabetes in our study included both classical type 1 diabetes at early-onset age and latent autoimmune diabetes in adults (LADA), a subtype of type 1 diabetes at late-onset age, therefore, the patients with type 1 diabetes in our study was more comparable to those with type 2 diabetes. Otherwise, if merely including classical type 1 diabetes, it can lead to the wrong conclusions. Thirdly, ketosis-onset diabetes in our study may not be fully equal to ketosis-prone diabetes, as data like PH and anion gap are not available in the present study, which identify whether patients have ketoacidosis or not. Finally, this was merely a single-center study with a relatively small number of patients, thus the multi-center studies in a larger sample should be performed to further clarify the atherosclerotic features of ketosis-prone diabetes.

## Conclusions

In conclusion, the prevalence and risk of lower limb atherosclerotic lesions in the ketosis-onset diabetes were remarkably higher than those of the control subjects without diabetes. The manifestations and risk factors of lower limb atherosclerotic plaque in the ketosis-onset diabetes were more similar to those in the non-ketotic type 2 diabetes rather than the type 1 diabetes. Therefore, the current study provided further clinical evidences to support the classification of ketosis-onset diabetes as a subtype of type 2 diabetes rather than idiopathic type 1 diabetes in adults.

## Abbreviations

BMI: Body mass index; WHR: Waist hip ratio; SBP: Systolic blood pressure; DBP: Diastolic blood pressure; LADA: Latent autoimmune diabetes in adults; TC: Total cholesterol; TG: Triglyceride; HDL-C: High density lipoprotein cholesterol; LDL-C: Low density lipoprotein cholesterol; Cr: creatinine; 24 h UAE: 24 h urinary albumin excretion rate; CCEs: Cardio-cerebrovascular events; LLDs: Lipid-lowering drugs; AHDs: Antihypertensive drugs; eGFR: Estimated glomerular filtration rate; FPG: Fasting plasma glucose; 2 h PPG: 2 h postprandial plasma glucose; FCP: Fasting C-peptide; 2 h CP: 2 h C-peptide; FIMT: Femoral intima-media thickness; PSV: Peak systolic velocity; OR: Odd ratio; 95% CI: 95% confidence interval; Exp (B): Expected (B).

## Competing interests

The authors declare that they have no competing interests.

## Authors’ contributions

LXL and WPJ designed the study, supervised the work, and reviewed and edited the manuscript. MFL, YR, and CCZ researched data, performed statistical analysis and wrote the manuscript. RZ, FL, JXL, YFT, WJZ, and YQB researched data and reviewed the manuscript. All authors read and approved the final manuscript.
